# A High-Fidelity Artificial Urological System for the Quantitative Assessment of Endoscopic Skills

**DOI:** 10.3390/jfb13040301

**Published:** 2022-12-16

**Authors:** Do Yeon Kim, Xiangzhou Tan, Moonkwang Jeong, Dandan Li, Arkadiusz Miernik, Tian Qiu

**Affiliations:** 1 Cyber Valley Group—Biomedical Microsystems, Institute of Physical Chemistry, University of Stuttgart, 70569 Stuttgart, Germany; 2 Department of General Surgery, Xiangya Hospital, Central South University, Changsha 411000, China; 3 Department of Urology, Faculty of Medicine, Medical Center—University of Freiburg, 79106 Freiburg, Germany

**Keywords:** additive manufacturing, organ phantoms, surgical simulation, endoscopy, data sensing in bio-models

## Abstract

Minimally-invasive surgery is rapidly growing and has become a standard approach for many operations. However, it requires intensive practice to achieve competency. The current training often relies on animal organ models or physical organ phantoms, which do not offer realistic surgical scenes or useful real-time feedback for surgeons to improve their skills. Furthermore, the objective quantitative assessment of endoscopic skills is also lacking. Here, we report a high-fidelity artificial urological system that allows realistic simulation of endourological procedures and offers a quantitative assessment of the surgical performance. The physical organ model was fabricated by 3D printing and two-step polymer molding with the use of human CT data. The system resembles the human upper urinary tract with a high-resolution anatomical shape and vascular patterns. During surgical simulation, endoscopic videos are acquired and analyzed to quantitatively evaluate performance skills by a customized computer algorithm. Experimental results show significant differences in the performance between professional surgeons and trainees. The surgical simulator offers a unique chance to train endourological procedures in a realistic and safe environment, and it may also lead to a quantitative standard to evaluate endoscopic skills.

## 1. Introduction

Minimally invasive surgical endoscopy is becoming prevalent across many other procedural and operative specialties, for example, in the department of urology. Since the first visualization of the upper urinary tract in 1929 [1,2], the use of flexible ureteroscopy (fURS) dramatical increased, for instance, by 83% from 1994 to 2004 [3]. However, postoperative complications (i.e., tissue wall injury, bleeding, and infection) following ureteroscopy procedures are not negligible [4,5] and constitute a serious public health problem [6]. It is, therefore, important to increase the surgeon’s experience to decrease the risk of severe complications of fURS [7]. Surgical training on surgical simulators in vitro is thus required by clinicians with hands-on opportunities to practice procedures and devices in a safe environment.

Current biomodels for the training of flexible endoscopy can be categorized into four major types [8]: cadaveric models [9], animal models [10], virtual reality models [11] and bench models [12]. Due to the accurate anatomical structures, the cadaver is considered a paragon for medical education. However, the scarcity of human cadaver resources and the preservation method of fresh human cadavers are still problematic [13,14]. Animal models show advantages as they exhibit real biological tissue characteristics; however, the deficiency in human anatomical structures and ethical issues cannot be ignored. Although VR models have promising prospects in surgical simulations, the limited haptic feedback and shortage of physical interactions with surgical tools make it unrealistic so far. The physical bench models play an important role in surgical training, which provides possible abstract anatomy demonstration and physical interactions [15,16]. For instance, Villa et al. developed a low-cost and portable box model (K-box) for the training of fURS and endourology [17]. AI-Jabir et al. presented an Advanced Scope Trainer (AST, Mediskills Limited, Northampton, UK), which allows the training of both basic and advanced procedures [18]. However, there still remain challenges in developing a realistic artificial organ phantom system with detailed anatomy, providing realistic imaging results as well as enabling quantitative feedback on the surgical performance to the trainees [19,20].

In our previous work, we reported high-fidelity soft organ phantoms fabricated by 3D printing and soft materials molding technologies, including a kidney phantom [21], a prostate phantom [22] and a liver phantom [23]. These technical advances provide the possibility to make realistic organ phantoms using biomimetic soft materials and, in the meantime, offer advanced data sensing compatibility to provide quantitative standards to evaluate the surgical performance—for example, the resection accuracy in an electrocautery surgery [22] by ultrasound imaging, and the precision of needle punctures in a transhepatic puncture procedure [23]. However, to the best of our knowledge, there is currently no bench-top endoscopic simulation system that can provide quantitative feedback to the trainees.

In this paper, we report the first high-fidelity endourological simulator for the training of fURS with the capability of quantitatively evaluating endoscopic skills by an automated computer vision algorithm. The physical phantom consists of a cost-effective soft kidney phantom, which is made of commercial polymer materials and includes a detailed pelvicalyceal system and a ureter. The collecting system phantom replicates the real human kidney with high-resolution, and sub-millimeter vascular features are embedded on the inner walls of the renal collecting system, rendering a realistic surgical scene and providing optical features for the detection by computer vision algorithm. Endoscopic professionals and trainee groups were recruited to perform surgical procedures on the system. A customized image analysis-based computer algorithm was developed to quantitatively assess the endoscopic skills. The evaluating criteria clearly distinguish the differences between the professional and the trainee and offer useful feedback for both groups to improve their surgical skills.

## 2. Materials and Methods

### 2.1. Fabrication of the Endourological Organ Models

**Figure 1:**
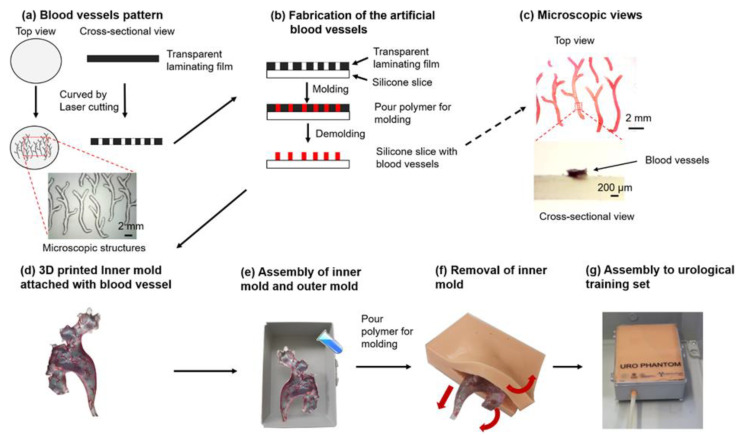
Fabrication process of the phantom of the kidney collecting system. (**a**,**b**) The fabrication of artificial blood vessels. This procedure includes vessel pattern printing and molding steps; (**c**) The microscopic images of the fabricated blood vessel; (**d**) The combination of the 3D printed inner mold and vascular structures; (**e**,**f**) Another molding procedure for the renal parenchyma fabrication; (**e**) The assembly of inner and outer molds; (**f**) Removal of the inner mold; (**g**) The phantom of the collecting system.

The fabrication process of the physical kidney collecting system model was based on a modified procedure of our previous work [21]. As illustrated in Figure 1, the process includes the steps of kidney collecting system 3D reconstruction, 3D printing the negative molds, and a two-step molding, which consists of the fabrication of the blood vessel features and the renal parenchyma.

During the 3D printing step, we utilized a 3D reconstructed digital model obtained by high-resolution computer tomography (CT) image data (with an iodinated contrast agent injected into the collecting system) in the previous study [21]. Based on the data, we designed the inner and outer molds that were used for the following two molding steps. The molds were printed on a 3D printer (Object260 Connex, Stratasys Ltd., Rehovot, Israel) with VeroClear^®^ material. In this study, we developed an additional step to extract and design vessel features on the kidney surface via endoscopy. In detail, we captured blood vessels exposed on the surface of the kidney collecting system and designed the vessel pattern (with the smallest feature width of 0.2 mm) in a software, Laser Dashboard™ (Epilog Laser, Golden, CO, USA). Then, the 2D vessel mold was carved on a transparent laminating cycloid film (R&B Laminiersysteme GmbH, Hollenstedt, Germany) using a laser cutter (Zing 16, Epilog Laser, Golden, CO, USA) as a mask. A silicone film of 0.8 mm in thickness (Ecoflex, Smooth-on Inc., Macungie, PA, USA) was placed underneath the laminating film.

The first molding step for vessel pattern fabrication was prepared with a red-pigmented silicone material (1 wt.% of Psycho Paint™ and Ecoflex, Smooth-on Inc., Macungie, PA, USA) filled through the grooves. Therefore, the blood vessel patterns were replicated onto the silicone film. The silicone slices with blood vessels pattern were cut into small pieces, attached to the surface of the inner mold and fixed with another thin layer of translucent silicone rubber, as shown in Figure 1b. The vessels were randomly oriented, and the location and density of the patterns were according to medical knowledge. A box (Polystyrene, inner size 15.1 cm × 11.0 cm × 4.5 cm) was used as an outer mold, and the inner mold with blood vessels was assembled with the outer mold (Figure 1d). For the second molding step, for renal parenchyma fabrication, a skin-colored silicone rubber material (1 wt.% of Psycho Paint TM and Ecoflex, Smooth-on Inc., Macungie, PA, USA) was poured into the assembled mold and cured in an oven at 65 °C for 4 h (Figure 1e). The collecting system phantom was then demolded from the outer mold, and the inner mold was removed via a 10 cm long incision from the side (Figure 1f). Additionally, a silicone tube (with an inner diameter of 6 mm, an outer diameter of 7 mm, and a length of 40 cm) was connected to the collecting system model to simulate the ureter. The joint and the incision were finally sealed with silicone adhesive (Smooth-on). The collecting system phantom was assembled into a box (40.0 cm × 30.0 cm × 17.0 cm) as the final endourological phantom, which resembles the position and dimension of the human urological system, as shown in Figure 1g.

### 2.2. Validation of the Phantom Using Endoscopy and CT

**Figure 2:**
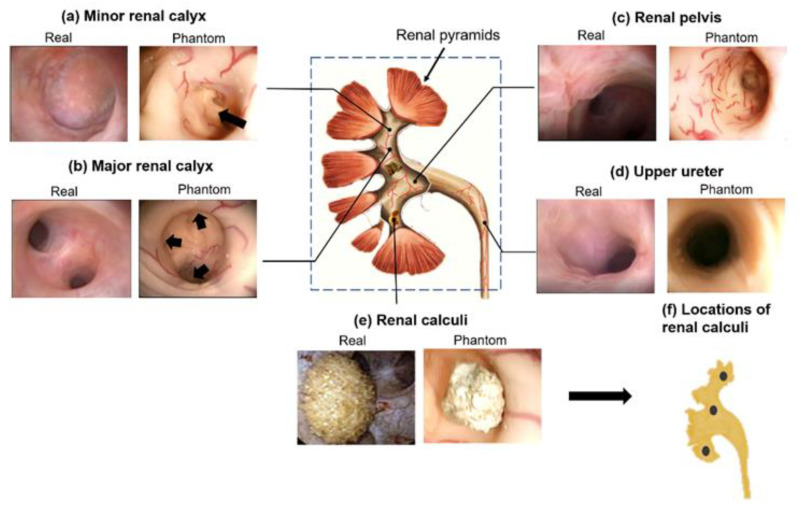
The validation of the soft endourological phantom using ureteroscopy. (**a**–**e**) Different anatomical sections are compared with real human endoscopic images. Black arrows in (**a**,**b**) refer to the renal papillae and minor calyces, respectively. The real renal calculus picture in (**e**) is obtained from [24] with permission. (**f**) Corresponding locations of three renal calculus in the proposed kidney model.

The collecting system phantom was validated using a flexible sensor ureterorenoscope (9.9 French, BOA Vision EF, Richard Wolf GmbH, Knittlingen, Germany). Prior to endoscope intubation, a ureteral access sheath (Flexor^®^ Ureteral Access Sheath, Cook Medical, Bloomington, IN, USA) was inserted in the ureter, and lubricant was applied to the endoscope surface for smooth insertion. Then, the inner surface of the collecting system in the phantom—i.e., physiological and pathological structures, including the ureter, the renal pelvis, the major renal calyx, the minor renal calyx, and the renal calculi—were observed by the endoscope (Figure 2). The obtained videos of our phantom model were compared with real patients’ endoscopic videos to evaluate detailed realistic representation. The recording and the use of the human endoscopic video in this study received the informed consent of the patient and were approved by the ethics committee of the Albert-Ludwigs University of Freiburg (under protocol number 567/15 with the amendment from 26 June 2018). The endoscopic image of real renal calculi was obtained from reference [24] with permission.

The 3D shape of the collecting system was validated by comparing two CT scans: the human cadaver kidney reference and the fabricated phantom. For the verification of the resemblance, we used CT scanning, similar to human kidney imaging, as reported in [21]. The CT scan (Somatom Force, Siemens, Erlangen, Germany) of the phantom was carried out without a contrast agent due to high image contrast between the silicone material of the phantom and the air. The shape of the collecting system in the phantom with the stones was 3D reconstructed and exported as a .stl file (InVesalius v3.1.1, Renato Archer Information Technology Center, Campinas, Brazil). The mesh of the collecting system phantom was then aligned and compared with that of the human collecting system by computing the point-to-point distances between the two cloud points in the software (CloudCompare v2.11, Telecom ParisTech and the R&D division of EDF, Paris, France). A pseudo-color image was displayed to show the spatial errors of the phantom compared to the original design as a reference.

### 2.3. Design and Prototyping of Surgical Endoscopic Simulator

**Figure 3:**
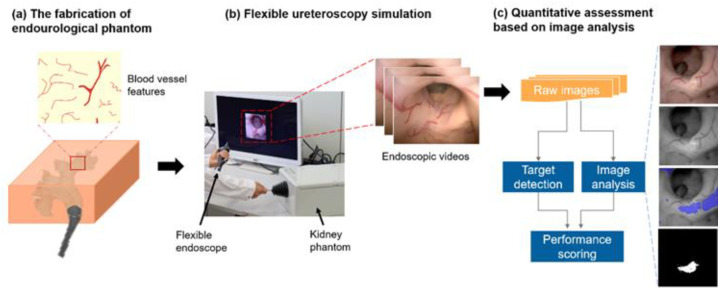
The schematics of an endourological phantom for the training of flexible ureteroscopy. (**a**) The kidney phantom was fabricated with the collecting system anatomy and the detailed vascular features; (**b**) Flexible ureteroscopy is simulated and endoscopic videos are recorded; (**c**) The performance of endoscopic skills is quantitatively and automated measured by the image analysis.

The purpose of this preliminary development was intended to (1) establish performance parameters and (2) measure and differentiate surgical skills (equivalent to parameters) into professional or trainee. As the first phase toward the goal, we collected endoscopic screening data of two groups—professional and trainee—using the fabricated kidney phantom (Figure 3). The professional group denotes physicians who have previously performed endoscopies more than 50 times, while the trainee group refers to medical students without such experience. Three artificial stones were placed in advance at the superior major calyx, the superior minor calyx, and the inferior major calyx, respectively. Each subject was required to endoscopically inspect these artificial stones. The procedure was repeated eight times for each group, respectively. All procedures were performed under the same experimental conditions (of organ phantom validation using endoscopy) as mentioned in Section 2.3, and the endoscopic videos were recorded (HD60 S+, Elgato, 60 FPS, 1080p) for the skill assessment processes.

### 2.4. Quantitative Assessment of the Endoscopic Skills by Video Analysis

To assess the endoscopic skills, a digital model was built with four metrics: (1) target detection, (2) fine movement, (3) visualization, and (4) efficiency. Endoscopy videos were acquired from both the professional and the trainee groups with the developed physical kidney model (see Section 2.3). All assessment methods were developed for image analysis of each endoscopy procedure.

The “Target Detection” is a parameter that relates to the ability of a complete examination, i.e., not to avoid any lesions in the endoscopy. Then, the rate is defined as:

(1) $$Target\;Detection=\frac{Detected\;Targets}{Total\;Targets}\times 100\%$$

where the Detected Targets refer to the number of renal calculi detected, and the total targets refer to the total number of renal calculi placed in the phantom.

**Figure 4:**
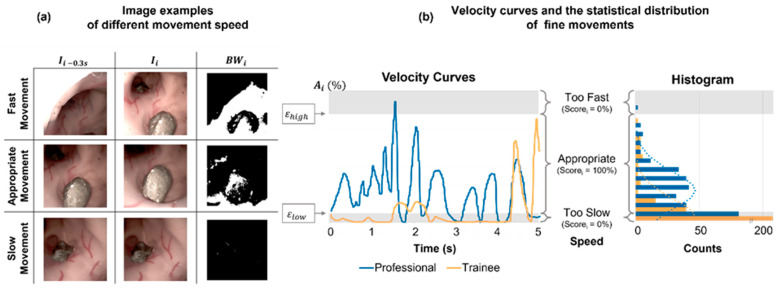
The assessment of the fine movement skill of endoscopy. (**a**) Examples of different moving speeds of the endoscope, white regions refer to those with high moving speeds. (**b**) Velocity curves of the fine movements and the histogram of the movement velocity distribution between the professional and the trainee (*p* < 0.01).

The “Fine Movement” represents the capability of endoscopic manipulation, i.e., the ability to correctly intubate and controllably maneuver the endoscope (Figure 4). This parameter computes the moving speed of the endoscopic tip, i.e., the difference between two frames with a time interval of 0.3 s. The parameter is calculated as follows:

(2) $$D_{i}\left(x,\;y\right)=\left|I_{i}\left(x,\;y\right)-I_{i-0.3\;s}\left(x,\;y\right)\right|$$

(3) $$BW_{i}\left(x,\;y\right)=\{\begin{array}{cc} 1, & D_{i}\left(x,\;y\right)>D_{Th}\\ 0, & D_{i}\left(x,\;y\right)\leq D_{Th} \end{array}$$

(4) $$A_{i}=\left(\frac{\sum_{x=1}^{n}\sum_{y=1}^{m}BW_{i}\left(x,y\right)}{Image\;Size}\right)\times 100\%$$

(5) $$Fine\;Movement_{i}=\{\begin{array}{cc} 100, & \varepsilon_{low}\leq A_{i}\leq \varepsilon_{high}\\ 0, & A_{i}<\varepsilon_{low}\;or\;A_{i}>\varepsilon_{high} \end{array}$$

(6) $$Fine\;Movement=\bar{Fine\;Movement_{i}}$$

where $D_{i}\left(x,\;y\right)$ represents the absolute difference between the two images and $I_{i}\left(x,\;y\right)$ is the pixel grayscale of *i*-th image at coordinates (x, y). $BW_{i}$ is a binary image with the threshold $D_{Th}$, where it was set as 50. $A_{i}$ is the coverage percentage of the whole frame, and the score of $Fine\;Movement_{i}$, is introduced with two cutoff values $\varepsilon_{low}$ and $\varepsilon_{high}$, i.e., only an appropriate moving speed gets the score, and either too slow or too fast gets zero point (see Figure 4b for the illustration). The overall score of the fine movement is an average of all frames in a video.

The “Visualization” represents the capability of acquiring effective information in the endoscopic vision field. For example, over-exposed regions do not provide any effective information, which should be avoided in the procedure. Furthermore, the contact of endoscopic tips with the wall of the urological tract could lead to severe complications, e.g., perforation, bleeding, and urinary infection. Over-exposure is often an indication that the endoscopic tip touches the wall, which should be avoided. Accordingly, the over-exposed area is defined as the areas with an intensity value larger than 245 (represented in white pixels on the second column, $BW_{i}$ images). The centroid and the size of each blob were calculated after the denoise step (MATLAB R2020b, Mathworks Inc., Natick, MA, USA). The distance $d_{i}$ was measured between the center coordinates (O) of the frame and the centroid (C) of the over-exposed region. Comparing the distance $d_{i}$ to the half diagonal length of the frame r, the weight function (w) is defined as:

(7) $$w=\{\begin{array}{cc} 1, & 0\leq d\leq r/3\\ 2/3, & r/3<d<2r/3\\ 1/3, & 2r/3\leq d\leq r \end{array}$$

And the score of visualization is defined as:

(8) $$Visualization=\bar{1-wA_{i}}\times 100\%$$

where $wA_{i}$ is the weighted area percentage of the $A_{i}$. For frames with multiple over-exposed blobs, the maximum value of the weighted area blob is chosen.

The “Efficiency” measures the time that a subject needs to complete a predefined task in the fURS procedure, and the parameter is defined as:

(9) $$Duration=\left(\frac{T_{max}-T}{T_{max}}\right)\times 100\%$$

(10) $$Efficiency=\bar{Target\;Detection+Duration}$$

where T refers to the time, and we set $T_{max}$, the maximum time allowed for the completion of the procedure, as 5 min in this study.

The full length of all videos from the two groups were analyzed. Statistical analysis was carried out in MATLAB using the Kolmogorov–Smirnov test (alpha = 0.05) for the normality test and the *t*-test (unpaired, two-tail) for the significance test, where *p* < 0.01 was considered as a significant difference. The histograms of the “Fine Movement” (Figure 4b) and the “Visualization” were calculated in Excel Visual Basic for Applications (VBA, 2016, Microsoft, Redmond, WA, USA) and fitted with a normal distribution.

## 3. Results

### 3.1. Endoscopic Validation of the Collecting System Phantom

The endourological phantom was successfully fabricated following the fabrication process in Figure 1. A flexible ureteroscope was used to visualize the inner structures and surfaces of the collecting system phantom. The anatomic structures, including the upper ureter, the renal pelvis, the major renal calyx, the minor renal calyx, and the calculi, were recorded and shown in Figure 2 (also see Video S1). They show very similar appearances, i.e., topology, colors and vascular structures, to the real human kidney.

Minor calyces are cup-shaped tubes that drain the urine from the triangular-shaped renal pyramids [25]. As shown in Figure 2a, the shape of the minor calyces in the phantom is well replicated that of a human kidney (the black arrow points to the renal papillae). Major calyces, including superior major calyces, middle major calyces, and inferior calyces, are formed by uniting a few minor calyces. Figure 2b shows the endoscopic image of the superior major calyces. Three minor calyces (black arrows) join to form the major calyx. The renal pelvis is a funnel-shaped sac, a joint between the major calyces and the ureter. In Figure 2c, the renal pelvis of the phantom also highly resembles that of a human kidney, as an expanded end of the excretory duct connected with major calyces. Figure 2d,e show the fidelity of the ureter and the calculi in the phantom.

The blood vessels are clearly visible in the regions of the minor renal calyx, major renal calyx and renal pelvis in the phantom (Figure 2a–c, Video S1). The fabrication method successfully fabricates vessels down to 0.39 ± 0.01 mm in width and 0.24 ± 0.01 mm in height (as shown in the microscopic images in Figure 1c). To the best of our knowledge, this is the first renal collecting system phantom with 3D vascular details at sub-millimeter resolution. The vasculatures not only render a more realistic scene for the endoscopic simulation but also offer strong features for automated image analysis for the quantitative assessment of endoscopic skills.

### 3.2. CT Validation of the Collecting System Phantom

**Figure 5:**
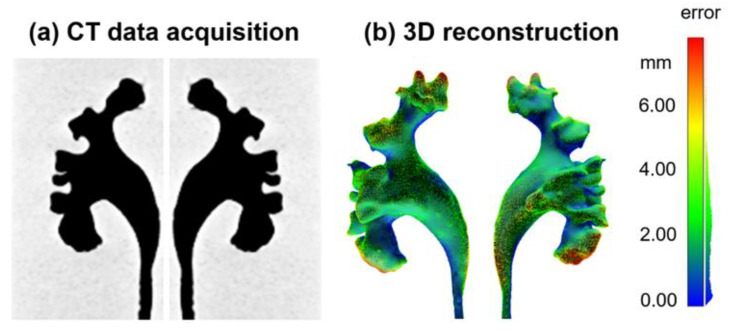
The validation of the anatomy of the collecting system phantom. (**a**) CT images of the phantom; (**b**) The quantitative error analysis of the phantom compared to the real human collecting system by a point cloud comparison.

The shape of the collecting system phantom was measured by a CT scan and 3D reconstructed to quantify the spatial error compared with the original 3D design. The quantitative evaluation of the distance differences between the human collecting system and the phantom shows a mean distance difference of 2.3 mm (mainly distributed in the range of 0.9–3.7 mm, Figure 5). The bounding box dimension of the collecting system is about 11.6, 6.0 and 3.5 cm in length, width and height, respectively. The collecting system phantom highly resembles the real human kidney, and the fabrication schemes of the phantom provide a high-resolution method to replicate 3D human organs. The largest spatial errors are mainly located in the upper zones of the superior renal calyx and the lower zones of the interior renal calyx. The other anatomical structures, e.g., the renal pelvis and major renal calyx, are well replicated.

### 3.3. Ureteroscopy Simulation and Quantitative Assessments of the Endoscopic Skills

**Figure 6:**
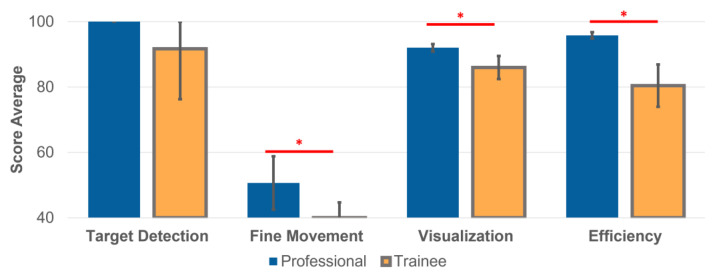
The overall scores of endoscopic skills, including target detection, fine movement, visualization, and efficiency. The error bars represent standard deviations, and * indicates statistical difference where *p* < 0.01.

The training of flexible ureteroscopy follows the workflow shown in Figure 3. Scores of the endoscopic skills were quantified with four metrics, i.e., the target detection, the fine movement, the visualization, and the efficiency, computed by automated image analysis. The videos of the two groups—the professional and the trainee—were analyzed on a workstation, and the results were obtained within several minutes; thus, one can expect the analyzed results are presented to the trainee right after each training session. The four average scores of the two groups are 100%, 51%, 92% and 97% for the professional, and 92%, 40%, 86%, and 80% for the trainee, respectively (Figure 6). The differences of the last three metrics are statistically significant (*p* < 0.01 for all three using unpaired *t*-test) respectively using the Wilcoxon rank sum test unpaired *t*-test) and passed the normality test (using Kolmogorov–Smirnov test).

To calculate the target detection rate, the participants were, therefore, requested to expose all stones in the fURS. The professional identified all three calculi; however, the trainee did not expose all three targets the first few times. The most difficult but common location of renal calculi is in the lower calyx. The renal calculi on this site can be identified only after retroflexing the scope in certain degrees due to the existence of the infundibulopelvic angle (IPA). Several studies have reported IPA is associated with difficulties of endoluminal surgery, and steep IPA can be considered as a factor that influences fURS-related complications, stone-free rate (SFR), length of operation and so on [26,27].

The two parameters—fine movement and visualization—also show significant differences between the two participating groups. Comparable to a fine movement example shown in Supplementary Materials Video S2, the trainee uses almost static and abrupt movements, as seen in the curve shown as the yellow curve in Figure 4b. On the contrary, the professional intubates the scope continuously with an appropriate speed, shown as the blue curve in Figure 4b. The fine movement scores for the professional in some time periods are also low, suggesting the movement should be slowed down to inspect potential lesions more carefully. Moreover, the visualization score and its distribution throughout each procedure remain high, with small variations for the professional, while fluctuating with high variations for the trainee (also see Supplementary Materials Video S3).

We observed the average examination duration of each group to compare the efficiency score. As a result, the trainee (15.4 min) spent almost three times longer than the professional (4.3 min) on average. According to experienced urologists, it is a general guideline that such an endourological procedure should be finished within 5 min. The trainee took too long and also missed renal calculi, especially at the beginning of trials. It clearly suggests the deficiency of the trainee in the procedure, which calls for more training.

## 4. Discussion

The age of traditional medical training—“see one, do one, teach one”—has changed in the last decades. There is an increasing tendency towards simulation-based training [28]. Our paper reports a soft, high-fidelity kidney phantom that exhibits hollow collecting system structures and vascular patterns based on the reconstructed data from a human kidney. Through video analysis, the assessment system can provide quantitative feedback for several metrics, including target detection, fine movement, visualization, and efficiency, to facilitate the trainees to adjust their learning strategy and sharpen their learning curves. Furthermore, the examinees with different experience levels show significant differences in the above parameters, which provide potential possibilities for an automatic marking process. Our study is a proof of concept that computer-aided video analysis offers objective measures to differentiate surgical skills, and it offers new possibilities for automated assessment of endoscopic skills by computers in a standardized fashion.

The fidelity of surgical simulators is essential to the training performance and the acceptability of simulation [29]. Our kidney phantom highly mimics the anatomical structure of the collecting system, including the minor renal calyx and renal papilla, which allows the training of fURS, especially those involving deep intubation into the renal calyx and papilla. Multi-modality medical imaging, including CT and endoscopy, were used for the validation of the phantom. As shown in the CT scan, the complex structures of the collecting system were accurately replicated that of the human kidney. The endoscopic images of the phantom also realistically imitate that of the human collecting system. In addition, the vascular patterns were attached to the inner cavities of the collecting system to simulate the vascular network on the surface of the mucosa. The vascular network not only increases the fidelity of the phantom but also improves the sense of spatial orientation for the surgeons. Additionally, it adds recognizable features to facilitate image analysis, which in return improves the efficacy of the performance feedback system.

The reported method is suitable for building physical biomodels for surgical simulation, especially for the training of flexible endoscopy. The physical model is reusable, as it is made of durable commercial polymer materials, which offer clear advantages over cadaveric or animal model-based surgical simulators. The fabrication process of a physical organ model currently takes approximately 2–3 h of labor in the lab, and the reported workflow allows automation and mass production in the future, which will reduce the cost making the model economical for surgical training. Moreover, the kidney phantom can be optimized in many aspects. The current model, made of silicone material, is watertight. It allows the simulation of the natural urine flow as well as the irrigation through the endoscope. The latter may lead to an undesired pressure increase in the collecting system, which should be avoided in endourological surgeries. In addition, the simulation of active physiological movements, for example, the respiratory movement of the kidney, can also be implemented in the future to create a more realistic simulation scene for endourological surgeries. Lastly, quantitative parameters can be further explored and determined to assess an individual’s surgical skills and provide specific, constructive feedback. In this study, we have proven this goal can be achieved with the proposed physical model.

## 5. Conclusions

We report the quantitative assessment of endoscopic skills based on analyzing the videos of surgical simulation in a high-fidelity phantom. The validation using CT and endoscopy shows the high fidelity of the collecting system phantom to a real human kidney. We also established four quantitative criteria in the digital model to evaluate the fURS performance, and the system revealed significant differences between the professional and trainee surgeons. Our work sheds light in the realistic and safe training of endoscopy as well as the objective and quantitative assessment of endoscopic skills.

## 6. Patents

A.M. has a patent WO 2017/207361 pending. T.Q. has a patent EP 3251811 issued and a patent WO 2017/207361 pending.

## Data Availability

Not applicable.

## Supplementary Materials

The following supporting information can be downloaded at: https://www.mdpi.com/article/10.3390/jfb13040301/s1, Video S1: An endoscopic video of the endourological phantom; Video S2: A video showing the video analysis of the parameter “Fine movement” by comparing the endoscopic videos of professional and trainee group; Video S3: A video showing the video analysis of the parameter “Visualization,” by comparing the endoscopic videos of professional and trainee group.
